# Primary Tumor Resection for Rectal Cancer With Unresectable Liver Metastases: A Chance to Cut Is a Chance for Improved Survival

**DOI:** 10.3389/fonc.2021.628715

**Published:** 2021-03-15

**Authors:** Jia-nan Chen, Sami Shoucair, Zheng Wang, Joseph R. Habib, Fu-qiang Zhao, Jun Yu, Zheng Liu, Qian Liu

**Affiliations:** ^1^Department of Colon and Rectal Surgery, National Cancer Center, National Clinical Research Center for Cancer, Cancer Hospital, Chinese Academy of Medical Sciences and Peking Union Medical College, Beijing, China; ^2^Department of Surgery, Johns Hopkins University School of Medicine, Baltimore, MD, United States

**Keywords:** rectal cancer, SEER (Surveillance Epidemiology and End Results) database, metastasis, liver, resection

## Abstract

**Background:** About half of the patients with rectal cancer will develop liver metastasis during the course of their illness. Unfortunately, a large proportion of these metastases are unresectable. Surgical resection of the primary tumor vs. palliative treatment in patients with unresectable synchronous liver metastases remains controversial.

**Methods:** Patients with rectal cancer with surgically unresectable liver metastases were identified from the Surveillance, Epidemiology, and End Results (SEER) database from January 1, 2010, to December 31, 2015. According to different treatment modalities, patients were divided into a primary tumor resection group and a non-resection group. Rates of primary tumor resection and survival were calculated for each year. Kaplan–Meier methods and Cox regression models were used to assess long-term survival. Multivariable logistic regression models were used to evaluate factors potentially associated with primary tumor resection.

**Results:** Among 1,957 patients, 494 (25.2%) had undergone primary tumor resection. Patients with primary tumor resection had significantly better 5-year survival rate (27.2 vs. 5.6%, *P* < 0.001) compared to the non-resection group. Chemoradiotherapy with primary site resection was associated with the longest mean and 5-year OS (44.7 months, 32.4%). The Cox regression analyses of the subgroup indicated that patients who underwent primary tumor resection had improved survival compared with those who did not undergo resection in all 25 subgroups. Factors associated with primary tumor resection were well or moderately differentiated tumor grade, undergoing radiation, and primary tumor size <5 cm.

**Conclusions:** The majority of patients with rectal cancer with unresectable liver metastases did not undergo primary tumor resection. Our results indicate that resection of the primary tumor appears to offer the greatest chance of survival. Prospective studies are needed to confirm these results.

## Introduction

Colorectal cancer (CRC) is the third most common cancer worldwide and is associated with a high mortality rate (1). Distant metastasis is the leading cause of cancer-related mortality with the liver constituting the most common site of distant metastases. In fact, ~20% of patients suffer from liver metastases at the time of diagnosis, whereas about 50% of patients develop liver metastases during the course of their illness (2, 3). Liver resection combined with chemotherapy is the only treatment offering the possibility of long-term survival in patients with metastatic colorectal cancer (mCRC) and can lead to a 5-year survival rate of 40–50% and 10-year survival rate of 20% (4, 5). Unfortunately, up to 80% of mCRC patients have an unresectable tumor and undergo palliative treatment as a standard of therapy (6).

In clinical practice, surgical resection of the primary tumor site in patients with unresectable liver metastases is recommended as a palliative approach. Initial resection of the primary tumor has been advocated to prevent malignancy-related complications such as bowel obstruction or perforation (7). Some studies have reported that resection of the rectal tumor at the primary site was independently associated with a better overall survival (7, 8). Conversely, other researchers reported that the benefits of primary tumor resection on survival are unclear since surgical resection of the primary tumor cannot eradicate the tumor completely (9, 10). Furthermore, surgery may delay the start of systemic chemotherapy, which may have a negative impact on survival (9, 10).

According to the National Comprehensive Cancer Network (NCCN) guidelines, the treatment of metastatic colon and rectal cancer is not uniform. This reflects the difference in anatomical, functional and metastatic patterns of the two entities (11). Although it has been established that the application of radiotherapy in metastatic rectal cancer can lead to better local control of disease prior to surgery, no role for radiation in metastatic colon cancer has been identified (12).

Despite the NCCN recommendation of the use of systemic chemotherapy or palliative care for mCRC patients with an asymptomatic primary tumor, previous study analysis of the SEER database showed that 67.4% of patients with stage IV CRC had undergone primary tumor resection (13). The study included mCRC patients diagnosed between 1988 and 2010, and their results showed that the resection rate was decreasing but survival rate improved. This serves to show that the role of surgery in the course of treatment for patients with advanced stage disease is an evolving field of study. A recent study by Concors et al. (14) evaluated the role of combined proctectomy and hepatectomy in patients with stage IV rectal adenocarcinoma. A stratified analysis was able to identify the role of combined therapy in offering improved survival in a specific cohort of patients with metastatic rectal adenocarcinoma. Although colon and rectal cancer have different treatment strategies, no multicenter, prospective clinical trial has evaluated the value of resection of the primary tumor for patients presenting with unresectable metastatic rectal cancer. The primary goal of this study was to explore the primary tumor resection rate in patients with unresectable metastatic rectal cancer and to assess the effect of resection on OS.

## Methods

### Data Resources

We obtained the rectal cancer data from the National Cancer Institute (NCI) linked Surveillance, Epidemiology, and End Results (SEER) database. The SEER database contains demographic information and data regarding cancer incidence and survival from 18 population-based registries that represent ~30% of the US population. SEER is an open public database. Data related to patients are de-identified, therefore, there was no need for written informed consent for this study. The Institutional Review Board of the National Cancer Center, Chinese Academy of Medical Sciences approved this study.

### Study Population

**Figure 1:**
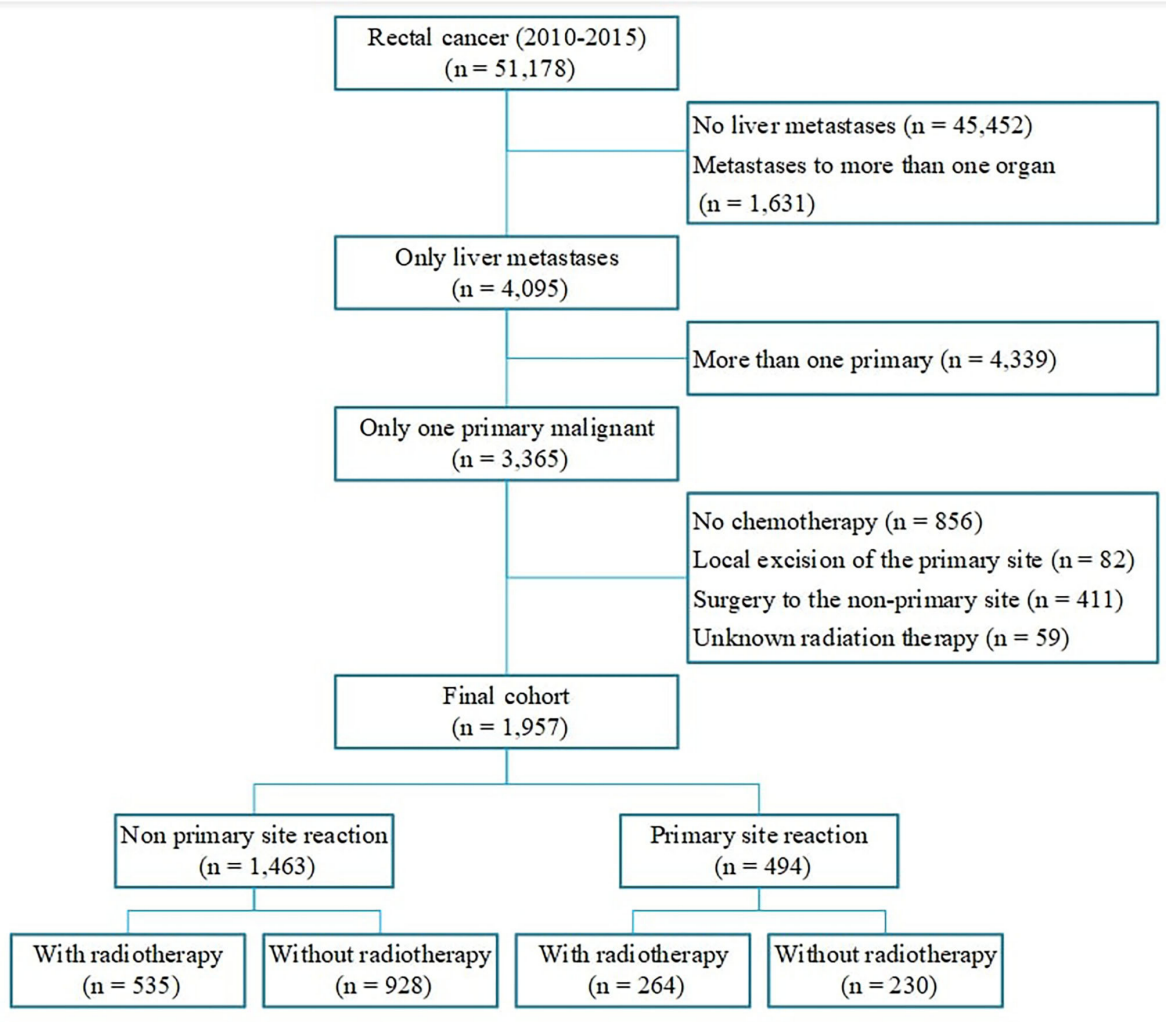
Flowchart of the inclusion and exclusion criteria.

Patients with rectal cancer with unresectable liver metastases diagnosed between January 1, 2010, and December 31, 2015 were eligible to be included in the study. We included only patients with tumor sequence numbers labeled “one primary only,” patients Mets at diagnoses-Liver labeled “yes,” and patients with Collaborative Stage (CS) Mets at Diagnoses labeled “metastases limited to a single distant organ” or “staged as M1a.” Systemic chemotherapy is the standard treatment approach for patients with stage IV rectal cancer, therefore only patients who received chemotherapy were included in the study. We restricted the Surgery Primary Site to (1) no surgery of primary site; (2) partial proctectomy, such as low anterior resection, Hartmann's operation, total mesorectal excision; (3) total proctectomy (abdominoperineal resection). We excluded patients who underwent local excision of their tumor or local tumor destruction. Patients with unknown radiation therapy or radioactive implants were excluded. Patients who had surgery to the metastatic site were also excluded from our study. After excluding 49,221 patients who were not eligible, 1,957 cases were included in the final cohort. Patients were divided into the following two groups according to the treatment strategy of the primary site: (1) Patients with primary tumor resection; (2) Patients without primary tumor resection. Each group comprised two subgroups based on whether they received radiation (Figure 1). Other relevant clinical characteristics including age, race, gender, marital status, tumor size, tumor grade, year of diagnosis were also collected.

### Statistical Analysis

Baseline characteristics of patients with unresectable metastatic rectal cancer who had or had not undergone primary tumor resection were compared using the Chi-squared test. The primary tumor resection rate was calculated for each year from 2010 to 2015. Our primary outcome was the OS. OS was defined as the time in months from diagnosis to either death or the last follow-up date. Survival analysis was performed by year of diagnosis and treatment modalities. The survival probability was estimated by the Kaplan-Meier methods, and the differences in survival of different groups of patients were compared by using Log-rank tests. Univariate and multivariate Cox's proportional hazard regression models were performed to estimate the independent prognostic factors. We also used a multivariate logistic regression model to identify factors associated with primary tumor resection. To better evaluate the impact of primary tumor resection on the survival of patients, we then divided the patients into 25 subgroups, the subgroup analyses of OS were separately performed using Cox's regression model. All statistical tests were two-sided and statistical significance was defined as *P* < 0.05. All statistical analyses were performed using the SPSS statistical software package (version 21.0; Chicago, IL) and R software (version 3.6.3; www.r-project.org).

## Results

### Patient Characteristics

A total of 1,957 patients met our inclusion criteria (Figure 1), with a mean age of 58.87 ± 12.33 years. Overall, 25.2% of patients with unresectable metastatic rectal cancer had undergone primary tumor resection. At the time of presentation, patients were more likely to have been male, with an age of 50–75 years. Furthermore, patients who had undergone primary site resection were more likely to have been younger, white, and married compared with patients who had not undergone primary tumor resection. The current study also showed that patients with well-differentiated or moderately differentiated tumors, tumor size <5 cm and had undergone radiation were more likely to undergo primary tumor resection (Table 1).

**Table 1:** Baseline characteristics of unresectable metastatic rectal cancer patients between January 1, 2010 and December 31, 2015.

**Characteristics**	**All patients**	**Primary tumor resection**	**Non-resection**	***P-*value**
	**(*n* = 1,957)**	**(*n* = 494)**	**(*n* = 1,463)**	
Age at diagnosis, year, No. (%)	58.87 ± 12.33			0.012
21–49	428 (21.9%)	121 (24.5%)	307 (21.0%)	
50–75	1,327 (67.8%)	338 (68.4%)	989 (67.6%)	
76–96	202 (10.3%)	35 (7.1%)	167 (11.4%)	
Sex, No. (%)				0.119
Female	638 (32.6%)	147 (29.8%)	491 (33.6%)	
Male	1,319 (67.4%)	347 (70.2%)	972 (66.4%)	
Race, No. (%)				0.013
White	1,543 (78.8%)	399 (80.8%)	1,144 (78.2%)	
Black	221 (11.3%)	39 (7.9%)	182 (12.4%)	
Asian or Pacific Islander	188 (9.6%)	56 (11.3%)	132 (9.0%)	
Unknown	5 (0.3%)	0 (0.0%)	5 (0.3%)	
Marital status, No. (%)				0.001
Married	1,004 (51.3%)	288 (58.3%)	716 (48.9%)	
Single	452 (23.1%)	96 (19.4%)	356 (24.3%)	
Separated, divorced, or widowed	501 (25.6%)	110 (22.3%)	391 (26.7%)	
Radiation, No. (%)				<0.001
Yes	799 (40.8%)	264 (53.4%)	535 (36.6%)	
No	1,588 (81.1%)	230 (46.6%)	928 (63.4%)	
Tumor grade, No. (%)				<0.001
Well + Moderate	1,157 (59.1%)	369 (74.7%)	788 (53.9%)	
Poor + Undifferentiated	363 (18.5%)	84 (17.0%)	279 (19.1%)	
Unknown	437 (22.3%)	41 (8.3%)	396 (27.1%)	
Tumor size, cm, No. (%)				<0.001
0–5	648 (33.1%)	271 (54.9%)	377 (25.8%)	
>5	562 (28.7%)	155 (31.4%)	407 (27.8%)	
Unknown	747 (38.2%)	68 (13.8%)	679 (46.4%)	
Year of diagnosis, No. (%)				<0.001
2010	308 (15.7%)	100 (20.2%)	208 (14.2%)	
2011	309 (15.8%)	71 (14.4%)	238 (16.3%)	
2012	325 (16.6%)	84 (17.0%)	241 (16.5%)	
2013	316 (16.1%)	90 (18.2%)	226 (15.4%)	
2014	336 (17.2%)	56 (11.3%)	280 (19.1%)	
2015	363 (18.5%)	93 (18.8%)	270 (18.5%)	

### Primary Tumor Resection Rate by Year

**Figure 2:**
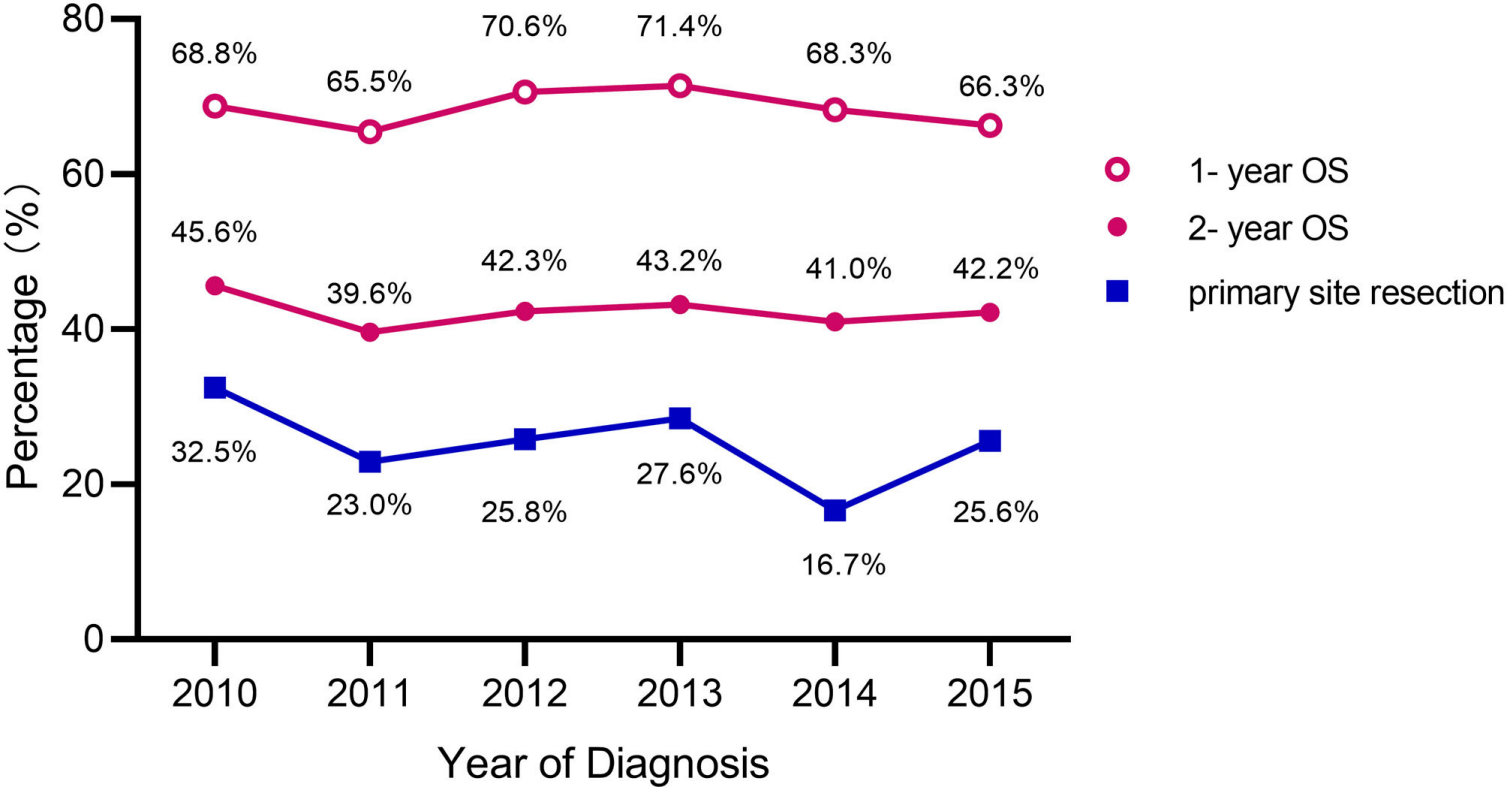
Primary tumor resection rates and OS for rectal cancer patients with unresectable liver metastases.

Figure 2 shows the primary tumor resection rates, 1-year OS, and 2-year OS by year. The highest resection rate was seen in 2010 (32.5%) and the lowest in 2014 (16.7%). The highest 1-year OS rate was seen in 2013 (71.4%) with a resection rate of 27.6%. 2010 had the highest 2-year OS rate (45.6%). Additionally, 2011 exhibited the lowest 1-year OS (65.5%) and 2-year OS (39.6%) with a primary tumor resection rate of 23.0%. As can be seen in the line chart, the 2-year OS change trend is basically consistent with that year of primary tumor resection rates.

### Survival Analysis

**Figure 3:**
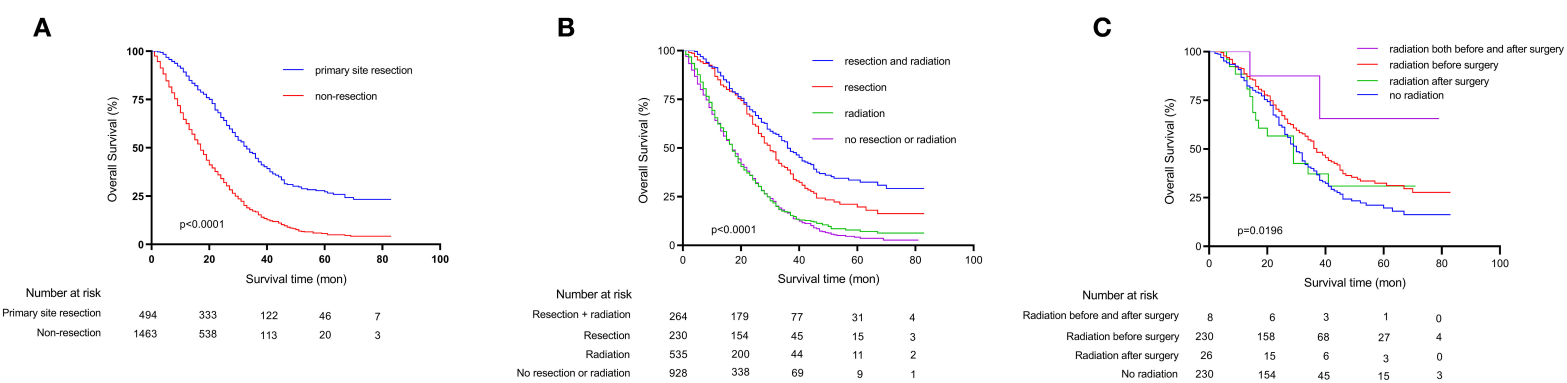
Kaplan-Meier survival curves depicting survival in unresectable metastatic rectal cancer patients. **(A)** Patients OS based on whether primary tumor was surgically resected or not. **(B)** Patients OS based on detailed treatment modality. **(C)** Patients OS based on radiation sequences.

The OS of the patients with unresectable metastatic rectal cancer were analyzed by using Kaplan-Meier survival curves, and the results are shown in Figure 3, Supplementary Table 1. Patients with primary tumor resection had significantly better 5-year OS compared to patients without primary tumor resection (*p* < 0.0001) (5-year OS: 27.2 and 5.6%, respectively) (Figure 3A). The mean survival in the two groups were 41.1 and 21.7 months, respectively. We further conducted a stratified analysis by whether patients underwent radiotherapy or not (Figure 3B). The results showed that patients who receive neither primary tumor resection nor radiotherapy had the worst 5-year OS rate (3.6%). Moreover, we analyzed the OS of different radiation sequences with surgery in the primary tumor resection group (Figure 3C), and the *P*-value of the log-rank test was 0.0196. However, when we further included this variable into multivariate Cox's regression analyses (Table 2), this difference was not significant (*P* = 0.055).

**Table 2:** Univariate and Multivariate analyses for OS of all patients (*n* = 1,957).

**Characteristics**	**Univariate analysis**		**Multivariate analysis**	
	**HR [95% CI]**	***P* value**	**HR [95% CI]**	***P-*value**
**Age at diagnosis, year**				
21–49	1		1	
50–75	1.174 (1.031-1.337)	0.016	1.129 (0.990–1.288)	0.069
76–96	1.771 (1.465–2.141)	<0.001	1.658 (1.366–2.013)	<0.001
**Sex**				
Female	1			
Male	0.947 (0.848–1.056)	0.328		
**Race**				
White	1		1	
Black	1.245 (1.065–1.455)	0.006	1.164 (0.993–1.363)	0.061
Asian or Pacific Islander	0.994 (0.834–1.185)	0.945	0.984 (0.824–1.174)	0.854
Unknown	0.498 (0.124–1.994)	0.325	0.431 (0.107–1.732)	0.236
**Marital status**				
Married	1		1	
Single	1.328 (1.170–1.508)	<0.001	1.281 (1.126–1.458)	<0.001
Separated, divorced, or widowed	1.278 (1.129–1.446)	<0.001	1.155 (1.019–1.309)	<0.001
**Tumor grade**				
Well + Moderate	1		1	
Poor + Undifferentiated	1.881 (1.651–2.144)	<0.001	1.853 (1.624–2.113)	<0.001
Unknown	1.393 (1.228–1.580)	<0.001	1.162 (1.022–1.322)	0.022
**Tumor size, cm**				
0–5	1		1	
>5	1.276 (1.117–1.458)	<0.001	1.115 (0.974–1.277)	0.115
Unknown	1.440 (1.273–1.628)	<0.001	1.039 (0.913–1.183)	0.562
**Year of diagnosis**				
2010	1			
2011	1.114 (0.943–1.315)	0.206		
2012	0.954 (0.806–1.129)	0.581		
2013	0.976 (0.821–1.160)	0.781		
2014	1.098 (0.919–1.312)	0.304		
2015	1.114 (0.912–1.361)	0.288		
**Treatment modality**				
Surgery + radiation	1		1	
Surgery	1.348 (1.069–1.700)	0.012	1.257 (0.995–1.588)	0.055
Radiation	2.647 (2.184–3.207)	<0.001	2.397 (1.969–2.918)	<0.001
None	2.819 (2.349–3.383)	<0.001	2.619 (2.168–3.162)	<0.001

Univariate and multivariate Cox's regressions were used to analyze the factors that may influence the OS (Table 2). Variables with *P* < 0.10 in the univariate analysis, including age at diagnosis, race, marital status, tumor size, tumor grade, treatment modality, were taken forward to multivariate Cox's regression analysis. Consequently, age older than 75 years at diagnosis (Hazard Ratio [HR] = 1.658; 95% confidence interval [CI]:1.366–2.013; *P* < 0.001), single (HR = 1.281, 95% CI: 1.126–1.458; *P* < 0.001), separated, divorced, or widowed (HR = 1.155, 95% CI: 1.019–1.309; *P* < 0.001), poorly differentiated or undifferentiated tumor (HR = 1.835, 95% CI: 1.624–2.113; *P* < 0.001), no primary site resection (radiation only or no radiation) (HR = 2.397, 95% CI: 1.969–2.918, *P* < 0.001; HR = 2.619, 95% CI: 2.168–3.162, *P* < 0.001, respectively) were confirmed to be independent risk factors for poor prognosis.

**Figure 4:**
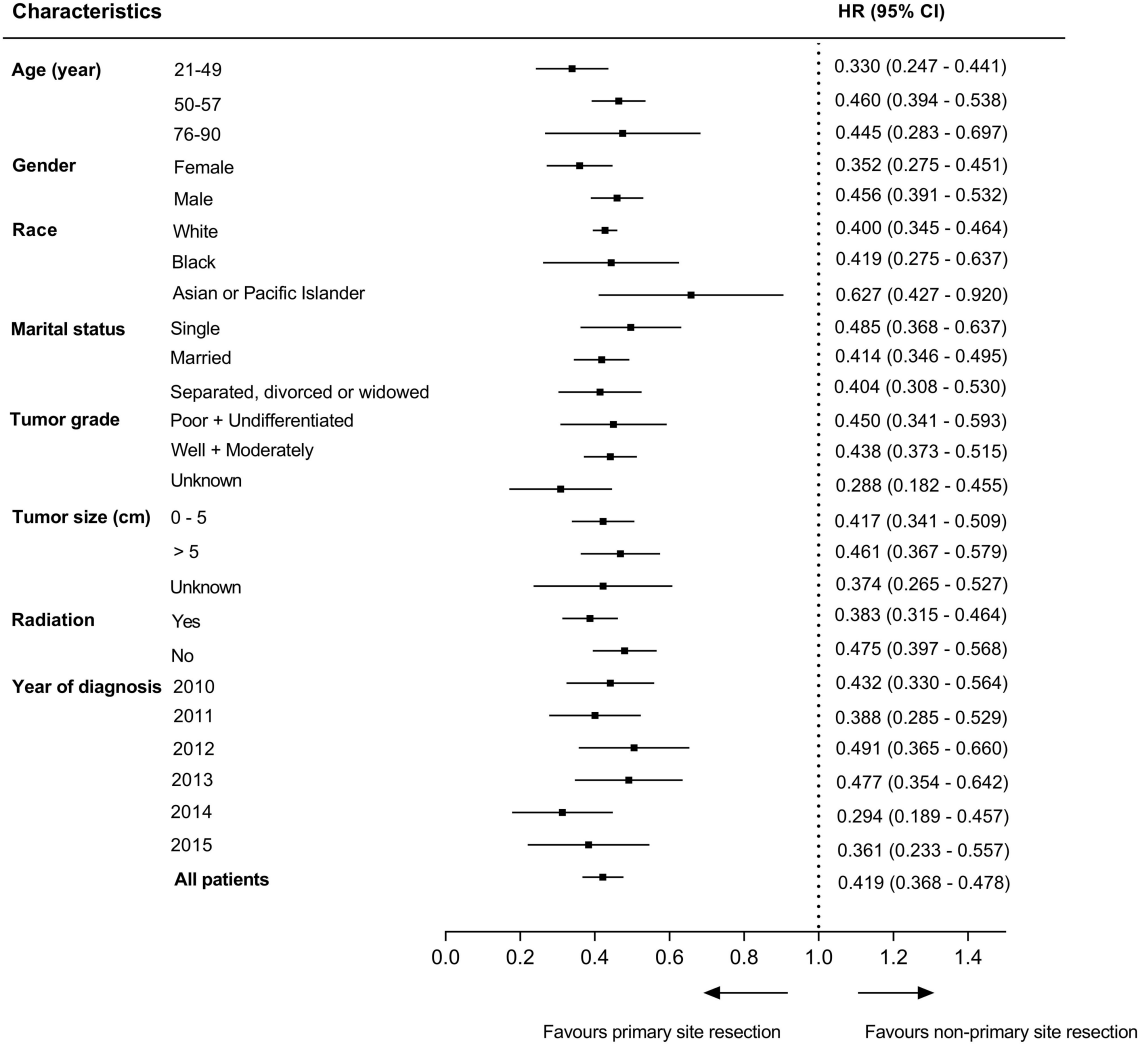
Survival comparisons between primary tumor resection group and non-resection group in subgroup analysis.

To better elucidate the effect of different treatment modalities on the prognosis of patients with unresectable metastatic rectal cancer, we divided patients into 25 subgroups according to demographic data and clinicopathological characteristics, Cox's regression model was used in each subgroup to estimate hazard rate and 95% confidence interval. The results indicated that patients who received primary tumor resection had a better prognosis than those who did not in all subgroups (*P* < 0.05) (Figure 4).

### Multivariable Analysis

A multivariable analysis was performed using logistic regression to determine factors associated with primary tumor resection at diagnosis. The results showed that having a well-differentiated or moderately differentiated tumor, receiving radiation, and tumor size ≤5 cm were significantly associated with primary tumor resection (all *P* < 0.001). On the other hand, patients who were diagnosed in 2012 and 2014 were less likely to have undergone surgical resection (Table 3).

**Table 3:** Multivariable analysis of factors associated with receiving primary tumor resection at diagnosis.

**Characteristics**	**OR (95% CI)**	***P*-value**
**Age at diagnosis, year**		
21–49	1 [Reference]	
50–75	0.935 (0.715–1.224)	0.627
76–96	0.636 (0.399–1.014)	0.057
**Sex**		
Female	1 [Reference]	
Male	1.116 (0.872–1.428)	0.383
**Race**		
White	1 [Reference]	
Black	0.732 (0.486–1.076)	0.11
Asian or Pacific Islander	1.220 (0.843–1.765)	0.291
Unknown	0 (0.000–)	0.999
**Marital status**		
Married	1 [Reference]	
Single	0.777 (0.581–1.040)	0.09
Separated, divorced or widowed	0.850 (0.642–1.125)	0.255
**Tumor grade**		
Poor + Undifferentiated	1 [Reference]	
Well + Moderate	1.502 (1.121–2.013)	0.006
Unknown	0.413 (0.271–0.631)	<0.001
**Radiation**		
No	1 [Reference]	
Yes	1.802 (1.438–2.257)	<0.001
**Tumor size, cm**		
>5	1 [Reference]	
0–5	1.750 (1.358–2.256)	<0.001
Unknown	0.283 (0.205–0.390)	<0.001
**Year of diagnosis**		
2010	1 [Reference]	
2011	0.707 (0.477–1.050)	0.086
2012	0.672 (0.459–0.983)	0.041
2013	0.821 (0.562–1.199)	0.307
2014	0.397 (0.264–0.596)	<0.001
2015	0.697 (0.481–1.010)	0.056

## Discussion

In this nationwide population-based study, we found that in patients with rectal cancer diagnosed with unresectable liver metastases, primary tumor resection was one of the strongest predictors of a better OS. The mean OS of patients receiving primary tumor resection was 41.1 months, which was almost 20 months longer than those without resection. Nevertheless, only 25.4% of the patients in our study underwent primary tumor resection between 2010 and 2015. Well-differentiated or moderately differentiated tumor grade, tumor size ≤5 cm, and having radiation are associated with an increased likelihood of having undergone primary tumor resection. Our findings also indicate that resection of the primary tumor was beneficial for patients with certain clinical and pathological characteristics namely those <75 years of age with well or moderately differentiated tumors. Although this is subject to the confounding effect of better tumor differentiation as the reason of the improved survival; however, this does not exclude the likely benefit of primary tumor resection in this specific population.

Whether resection of the primary tumor in patients with unresectable liver metastases affords a survival advantage is still controversial, and research in this area has remained rather limited. One previous study reported that the use of primary tumor resection in patients with stage IV CRC had been decreasing over time, the resection rates were 74.5% in 1988 and 57.4% in 2010, however, with the improvement of systemic chemotherapy, patient survival rates improved (13). Furthermore, the newly updated NCCN guidelines recommend against routine resection of the primary tumor (12). In our study, only patients with metastatic rectal cancer were enrolled. According to the year of diagnosis, the largest proportion of primary tumor resection occurred in 2010, where only 32.5% of patients underwent resection. The differences in the resection rates are most likely due to the fact that rectal surgery has a greater postoperative complication rate and frequently requires a diverting stoma, furthermore, abdominoperineal resection (APR) must be performed for the patients with low rectal cancer, making neither surgeons nor patients willing to receive surgery in a metastatic context. Although the study mentioned above suggested that patients' survival rates improved with a decreasing resection rate (13). However, the important limitations of this study are that the conclusion did not draw from the rigorous statistical method (no multivariate Cox's regression was performed) and they had no information about whether chemotherapy was received by patients, which makes it difficult to assess the relative contribution of resection and chemotherapy on outcomes.

There are some studies with findings that are consistent with ours. Venderbosch et al. performed a retrospective analysis of two phase III studies (CAIRO and CAIRO2) investigating the prognostic value of resection of the primary tumor in in patients with unresectable stage IV CRC. Their results indicated that resection of the primary tumor is a prognostic factor for median survival and progression-free survival in mCRC patients (8). They also reviewed the literature regarding this topic and identified 22 non-randomized, single-center studies, 14 of 24 studies demonstrated an improved median OS in the resection compared with the non-resection group. Matthieu et al. performed a study on the outcomes of 810 patients with CRC with unresectable synchronous metastases of which 59% underwent resection of the primary tumor. A lower baseline carcinoembryonic antigen (CEA), alkaline phosphatase levels, and normal white-blood-cell count (*P* < 0.001 each) was noted in the resection group when compared to the non-resection group. Primary tumor resection was independently associated with better OS (HR = 0.63, 95%CI: [0.53–0.75]; *P* < 0.001) (7).

The most important argument against an initial resection of the primary tumor is that surgery can delay the start of chemotherapy and patients are also subject to possible postoperative complications, both may have a negative effect on survival (8, 15). Scheer et al. reported that the overall postoperative morbidity in the patients with primary tumor resection ranged from 18.8 to 47.0%, which potentially delays beneficial systemic chemotherapy (16). Our results proved this partly true. While we analyzed survival based on the year of diagnosis, we found that the trend of 2-year OS was basically consistent with the resection rate, as the highest value of resection rate and 2-year OS both in 2010 (32.5 and 45.6%, respectively). However, the 1-year OS may be affected by surgery-related complications, and the trend was not as good as the former one (Figure 2). We speculated that postoperative complications have an impact on the 1-year OS, however, survival changes over time, primary tumor resection played a leading role in the 2-year OS.

The noted treatment difference between stage IV colon and rectal cancer is that radiotherapy is applied in metastatic rectal cancer for better local control of disease (17). Afshari et al. conducted a Swedish nationwide study to explore the prognostic factors that affect survival and their results showed that preoperative radiotherapy (*P* = 0.001), metastasectomy (*P* < 0.001) and radical resection of the primary tumor (*P* = 0.014) were better prognostic factors (18). From our results, we can see from the multivariate Cox's regression analysis that radiotherapy (HR = 1.257, 95%CI: [0.995–1.588]; *P* = 0.055) had no significant survival benefit for patients with metastatic rectal cancer, but patients who received radiotherapy were more likely to undergo primary tumor resection (OR = 1.802, 95%CI: [1.438–2.257]; *P* < 0.001). Furthermore, primary tumor resection is beneficial for survival in all subgroups of patients (25 subgroups, all *P* < 0.05). Therefore, radiotherapy might affect the OS indirectly.

Our study had several limitations. First, although we used multivariable analysis to adjust for clinical confounders in view of the difference between the primary tumor resection group and the non-resection group, it remains probable that primary tumor resection had been preferably performed in patients with better functional status, a selection bias cannot be excluded due to its retrospective nature and the lack of data on patient-specific comorbidities in the database. Second, the tumor size in the non-primary tumor resection group may go from endoscopic examination or computerized tomography (CT), so the values may not be as accurate as of the resection group, also key information like number and size of liver metastases were not recorded in the database; and the SEER database is short of detailed information about chemoradiotherapy regimen and biological targeted therapy, which could also influence the prognosis. Additionally, we only included patients diagnosed between 2010 and 2015, long-term survival data in those patients are still lacking, we observed only 2-year OS based on year of diagnosis, the survival trend might be more convincing if the follow-up time was longer.

## Conclusion

Our study demonstrates that primary tumor resection in patients with unresectable metastatic rectal cancer is associated with significant improvements in survival. However, only a quarter of the patients with metastatic rectal cancer received surgical resection of the primary site. Prospective, randomized trials are necessary to determine the role of primary tumor resection in patients with unresectable metastatic rectal cancer.

## Data Availability Statement

The original contributions presented in the study are included in the article/Supplementary Material, further inquiries can be directed to the corresponding author/s.

## Author Contributions

J-nC, SS, and ZW: acquisition of data. J-nC, JH, and F-qZ: analysis and interpretation of data. J-nC and SS: drafting of the manuscript. JY, ZL, and QL: critical revision of the manuscript. All authors reviewed the manuscript.

## Conflict of Interest

The authors declare that the research was conducted in the absence of any commercial or financial relationships that could be construed as a potential conflict of interest.
