# Supplementary material for: Primary Tumor Resection for Rectal Cancer With Unresectable Liver Metastases: A Chance to Cut Is a Chance for Improved Survival
Source: Front Oncol. 2021 Mar 15;11:628715. doi: 10.3389/fonc.2021.628715 (PMC8006931; doi:10.3389/fonc.2021.628715)
Supplement: Supplementary file 1 [file Table_1.DOCX]

| Table S1. Mean survival and 2-, 5- year OS of unresectable metastatic rectal cancer patients | | | |
| --- | --- | --- | --- |
|  |  |  |  |
| **Variables** | **Mean survival, mons** | **2- year OS** | **5- year OS** |
| Primary tumor resection | 41.1 | 65.60% | 27.20% |
| With radiation | 44.7 | 68.60% | 32.40% |
| Without radiation | 36.9 | 62.20% | 19.70% |
| Non-resection | 21.7 | 33.80% | 5.60% |
| With radiation | 23 | 33.50% | 7.10% |
| Without radiation | 21.1 | 34.00% | 3.60% |
| OS: overall survival | | | |
